# Editorial: Building capacity for sickle cell disease research and healthcare

**DOI:** 10.3389/fgene.2023.1226589

**Published:** 2023-07-12

**Authors:** Victoria Nembaware, Obiageli Eunice Nnodu, Raphael Zozimus Sangeda, Vivian Paintsil, Gaston Kuzamunu Mazandu, Nchangwi S. Munung, Ambroise Wonkam, Arturo J. Martí-Carvajal

**Affiliations:** ^1^ Division of Human Genetics, Department of Pathology, Faculty of Health Sciences, University of Cape Town, Cape Town, South Africa; ^2^ Centre of Excellence for Sickle Cell Disease Research and Training (CESRTA), University of Abuja, Abuja, Nigeria; ^3^ Department of Haematology and Blood Transfusion, University of Abuja, Abuja, Nigeria; ^4^ Department of Pharmaceutical Microbiology, Muhimbili University of Health and Allied Sciences, Dar es Salaam, Tanzania; ^5^ Directorate of Child Health-Komfo Anokye Teaching Hospital, Kumasi, Ghana; ^6^ Department of Child Health- School of Medicine and Dentistry, Kwame Nkrumah University of Science and Technology, Kumasi, Ghana; ^7^ Department of Genetic Medicine, Johns-Hopkins University School of Medicine, Baltimore, MD, United States; ^8^ Cochrane Centre, Facultad de Ciencias de la Salud Eugenio Espejo, Universidad UTE, Quito, Ecuador; ^9^ Cátedra Rectoral de Medicina Basada en Evidencia, Universidad de Carabobo, Valencia, Venezuela; ^10^ Faculty of Medicine, Francisco de Vitoria University, Madrid, Spain

**Keywords:** building capacity, sickle cell disease (SCD), research infrastructure, healthcare development, training, SCD knowledge advancement, SickleInAfrica, Africa

## 1 Introduction

Sickle cell disease (SCD) is the most frequent clinically significant genetic disorder that affects the production of hemoglobin, a protein found in red blood cells that carries oxygen throughout the body (Rees et al., 2010). SCD is particularly prevalent in Africa and the Americas. Although, according to the World Health Organization, SCD is a significant public problem, SCD is still classified as a neglected disease (Grosse et al., 2011). Since Africa has a high burden of SCD (Diallo and Guindo, 2014; Makani et al., 2020; Oron et al., 2020) and limited access to care and treatment, research studies and clinical efforts that will help to address the significant health burden of SCD are welcome.

On 02/07/2021, we launched a call for submissions to the Frontiers’ Topic series “Building Capacity for Sickle Cell Disease Research and Healthcare” to highlight ongoing projects, healthcare advances, advocacy and capacity building in the SCD field by inviting articles that address translational SCD research, standards of care for SCD patients globally, equitable access to improved and quality healthcare, and global or regional SCD research initiatives.

Nineteen manuscripts were submitted. After peer review, eleven and four manuscripts were accepted for publication in the Frontiers in Genetics and Frontiers in Pediatrics, respectively. These 15 articles discuss efforts to improve knowledge, the diagnosis, treatment, and management of the disease in African and American populations. Of these, ten are original articles describing SCD interventions. Three are research methodology papers, one is a brief research report and one is a case report.

Most of these articles discuss efforts to build research capacity for SCD in Africa. Nnodu et al. describe the efforts of the Sickle Pan African Research Consortium (SPARCo) with support from the Sickle Africa Data Coordinating Center (SADaCC) to develop skills and abilities for SCD research and healthcare services in Africa. The paper highlighted the training programs and workshops conducted by the consortium to enhance research capacity and improve healthcare services for SCD. About 1,726 participants attained skills development activities across the Ghana, Nigeria and Tanzania SPARCO sites. Skills have been enhanced in data management, SCD and research to underpin the core deliverables of SPARCo. Nkya et al. discuss the challenges of establishing a birth cohort for SCD research in Tanzania. The authors enrolled and followed -up visits of 341 babies with and without SCD. Out of these, 311, 186, 133, 81, 44, and 16 babies have returned for their 1st, 2nd, 3rd, 4th, 5th, and 6th visits, respectively. They collected demographic and clinical information for these babies because this platform may help understand the underlying mechanisms that influence early SCD manifestation and help devise intervention management which will reduce morbidity and mortality rates in children. Okeke et al. describe a new method for newborn screening for SCD in Nigeria using dried blood spots on the HemoTypeSC™ device. Of the 511 newborns from whom heel stick samples were collected (241 males and 270 females), HemoTypeSC using DBS identified 79.0% HbAA, 19.6% HbAS, 1.2% HbSS, and 0.2%) HbAC phenotypes. The isoelectric focusing (IEF) test identified 72.4% HbAA, 26.0% HbAS, 1.0% HbSS, and 0.6% HbAC phenotypes. The sensitivity, specificity, positive predictive value (PPV), negative predictive value (NPV) and overall accuracy of HemoTypeSC using DBS, compared to standard HemoTypeSC POCT, was 100%. Paintsil et al. report how SickleInAfrica’s initiative benchmarked to make recommendations for multi-level standards of care for SCD. The review yielded a guideline with recommendations for the management of SCD on 64 topics and subtopics for six different referral levels of healthcare, with the highest and lowest being the tertiary facilities and the home settings, respectively.

Other articles focus on specific aspects of SCD treatment and management. For example, Chianumba et al. evaluated the use of hydroxyurea (HU) in Nigeria and patients’ perceptions and experiences with the treatment. Of the 378 participants in this study, 89.7% were on HU, while 9 (10.3%) had stopped using HU. 92.5% of participants had fewer pain crises, 84.8% had fewer needs for blood transfusion, 86.3% had improved packed cell volume (PCV) and 84.6% had fewer hospital admissions due to HU use. Therefore patient-based advocacy is suggested to improve HU uptake and prioritization of HU availability and affordability. Treadwell et al. describe barriers to hydroxyurea use from the perspectives of healthcare providers, SCD patients and their families in the United States. Their results support strengthening provider understanding and confidence in implementing existing SCD guidelines and the importance of shared decision-making in addressing barriers to hydroxyurea use and combination therapies for SCD. Starosta et al. report on a case of infantile-onset Pompe disease complicated by SCD, highlighting the management challenges and considerations in such cases.

Other papers focus on the prevalence of SCD in different populations. Tutuba et al. report on the prevalence of hemoglobin-S and SCD baseline knowledge among 600 pregnant women attending antenatal clinics in Tanzania. They found a high prevalence of sickle cell trait and a low level of knowledge among the study participants but only 2 (0.3%) knew their SCD status. In comparison, 7.7% reported a family history of SCD. Martella et al. report on the distribution of HbS alleles and haplotypes in a multi-ethnic population in Guinea Bissau among 848 children (498 males and 350 females) and the implications for public health screening. The study found a high prevalence of the sickle cell trait and diverse sickle cell haplotypes in the study population, which has implications for public health screening in the region.

Ahmed et al. conducted a study on the patterns, outcomes, and predictors of pediatric medical admissions at Gadarif Hospital in Eastern Sudan. The study discovered that respiratory infections, malaria, and gastrointestinal infections were the three most common reasons for admission when reviewing 740 medical files, most (61.2%) of which were from males. The study also reported a high mortality rate of 5.7%, especially for babies, and malnourishment was the strongest predictor of death.

The SCD registry infrastructure and resources may help to improve SCD care and research in Africa. Some papers highlighted the efforts to create standards of care and databases for managing SCD. Nnodu et al. describe efforts to establish a database for SCD patient mapping and survival tracking in Nigeria using data from SPARCO among 7,767 people living with SCD at 25 health institutions across the six zones in Nigeria. Paintsil et al. report on establishing an SCD registry in Kumasi Ghana by the Sickle Pan-African Research Consortium in Kumasi, Ghana enrolling 3,148 SCD patients between December 2017 to March 2020. The article provides an overview of the challenges and successes of establishing the registry, including data Research Topic, quality control, and sustainability Research Topic.

Okocha et al. conducted a study on adiponectin and disease severity in sickle cell anemia patients attending a tertiary health institution in Nnewi, Southeast Nigeria. The study found that adiponectin levels were not significantly associated with disease severity and complications such as organ damage and acute chest syndrome. In their second paper, Okocha et al. conducted a cross-sectional survey on barriers to the therapeutic use of hydroxyurea for SCD in Nigeria. The study found that the most common obstacles were a lack of knowledge among healthcare providers, inadequate access to the medication, and concerns about its safety and efficacy. The study recommends interventions to improve awareness, access, and utilization of hydroxyurea for SCD in Nigeria.

Finally, Jonathan et al. conducted a study among 490 nurses and clinicians at Regional Referral Hospitals to investigate the knowledge and resource availability for SCD care in Dar es Salaam, Tanzania. The study found that most healthcare workers had inadequate knowledge about SCD and its management, with only 25.1% having a good knowledge of SCD. The study also found significant resource limitations, particularly regarding the availability of essential medications and laboratory tests. The study recommends the need for targeted educational programs and investments in healthcare infrastructure to improve SCD care in Tanzania.

The future of research direction from all these studies should inform interventions, including therapeutics that rely upon genomics and SCD markers (Wonkam, 2023).

## 2 Conclusion

The articles reviewed in this editorial highlight several challenges and opportunities in advancing SCD care and research in Africa. Key challenges include limited resources, inadequate knowledge and awareness, and insufficient research and clinical care infrastructure. However, there is hope, as demonstrated by the growing number of initiatives aimed at improving SCD care and research in Africa. To alleviate the challenges, there is a need for increased investment in SCD research and care, as well as greater collaboration and knowledge sharing among researchers, healthcare providers, policymakers and community stakeholders.

## 3 Recommendation

We recommend a coordinated and sustained effort to improve and increase capacity for SCD care and research in Africa, including increased funding for research and clinical care, expansion of newborn screening programs, and implementation of evidence-based guidelines for SCD management. Additionally, there is a need for increased community engagement and education to improve awareness and understanding of SCD and to address stigma and discrimination. Collaboration between researchers, healthcare providers, community stakeholders and policymakers is crucial to ensure that efforts are coordinated, aligned with the needs of affected communities and sustainable. By working together, key SCD stakeholders can make progress in advancing SCD care and research in Africa and ultimately improve the lives of those affected by this debilitating disease.
